# Spatial and Temporal Variations of Crop Fertilization and Soil Fertility in the Loess Plateau in China from the 1970s to the 2000s

**DOI:** 10.1371/journal.pone.0112273

**Published:** 2014-11-07

**Authors:** Xiaoying Wang, Yanan Tong, Yimin Gao, Pengcheng Gao, Fen Liu, Zuoping Zhao, Yan Pang

**Affiliations:** 1 College of Natural Resources and Environment, Northwest A&F University, Yangling, China; 2 Key Laboratory of Plant Nutrition and the Agri-environment in Northwest China, Ministry of Agriculture, Yangling, China; Institute of Botany, China

## Abstract

Increased fertilizer input in agricultural systems during the last few decades has resulted in large yield increases, but also in environmental problems. We used data from published papers and a soil testing and fertilization project in Shaanxi province during the years 2005 to 2009 to analyze chemical fertilizer inputs and yields of wheat (*Triticum aestivum* L.) and maize (*Zea mays* L.) on the farmers' level, and soil fertility change from the 1970s to the 2000s in the Loess Plateau in China. The results showed that in different regions of the province, chemical fertilizer NPK inputs and yields of wheat and maize increased. With regard to soil nutrient balance, N and P gradually changed from deficit to surplus levels, while K deficiency became more severe. In addition, soil organic matter, total nitrogen, alkali-hydrolysis nitrogen, available phosphorus and available potassium increased during the same period. The PFP of N, NP and NPK on wheat and maize all decreased from the 1970s to the 2000s as a whole. With the increase in N fertilizer inputs, both soil total nitrogen and alkali-hydrolysis nitrogen increased; P fertilizer increased soil available phosphorus and K fertilizer increased soil available potassium. At the same time, soil organic matter, total nitrogen, alkali-hydrolysis nitrogen, available phosphorus and available potassium all had positive impacts on crop yields. In order to promote food safety and environmental protection, fertilizer requirements should be assessed at the farmers' level. In many cases, farmers should be encouraged to reduce nitrogen and phosphate fertilizer inputs significantly, but increase potassium fertilizer and organic manure on cereal crops as a whole.

## Introduction

China has only 9% of the world's arable land and feeds nearly 22% of the world population [1]–[2]. This depends heavily on increasing grain production with the use of chemical fertilizers. Before the 1970s, farmers maintained the original agricultural practices, such as crop rotation, diversified plantation, manure application and legume crop integration, for soil fertility maintenance and pest and disease control. Since the late 1980s, the practice of applying organic manure in arable cropping systems has nearly come to an end [2]–[6]. From then on, almost all available organic manure has been used on vegetables and fruit trees, while the nutrients for cereal crops have been mainly in the form of chemical fertilizers. From 1970 to 2010, total annual grain production in China increased from 240 to 546 million tons (a 128% increase). However, inorganic fertilizer application increased from 3.51 to 55.62 million tons (a 1485% increase) over the same period [7].

Soil quality indicators are measurable soil properties that benefit food production or other specific functions, including physical, chemical and biological characteristics [8]. The increase or decrease in single soil index values, such as soil organic matter, total nitrogen and available nutrients, amplitude of variation and variation in time, can be used as a monitoring index for agricultural land management [9]–[11]. Given the spatial and temporal variation in characteristics of soil quality, it is necessary to compare or analyze two or more phase changes to understand the nature and mechanisms of soil quality [12].

Farmland fertilization is one of the most effective ways to maintain soil fertility and increase crop yields [13]–[15]. For this reason, information on household fertilization levels is of great value. In addition, wheat and maize are two of the most important food crops throughout the world, and they account for 51.7% of the total area for food crops and 53.5% of the total food production in 2010 in China [7]. Chemical fertilizer consumption data from official Chinese statistics do not contain information on usage for each kind of crop. It is imprecise to analyze and evaluate fertilizer efficiency using total amounts, because the distribution and application of fertilizer on specific crops are ambiguous [16].

Thus, the objectives of this study were to: (1) reveal the spatial and temporal variations of chemical fertilization and yields of wheat and maize at the farmers' level from the 1970s to the 2000s in the Loess Plateau in China; (2) reveal the spatial and temporal variations of soil fertility over the same period; and (3) reveal the relationships among fertilizer inputs, crop yields and soil fertility.

## Materials and Methods

### Ethics Statement

This study has been approved by the Agricultural Technology Extension Center of Shaanxi province, which is responsible for fertilization and soil fertility in Shaanxi province. All data in this study can be published and shared.

### Study area

**Figure 1:**
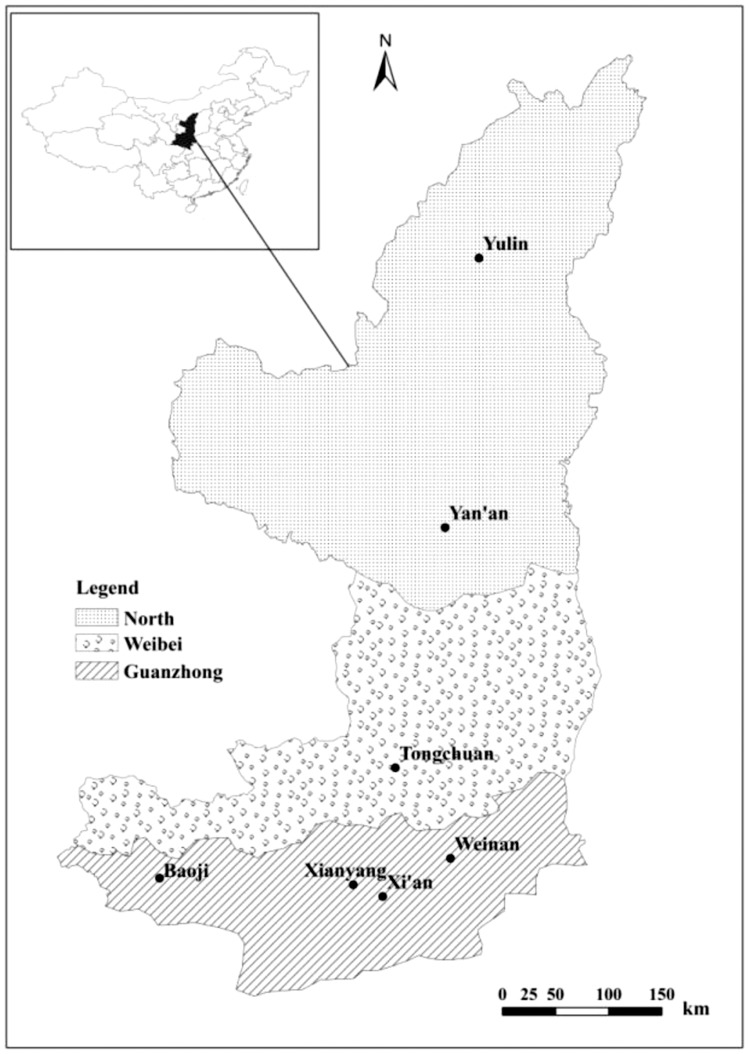
Map of the study area.

Shaanxi province (Figure 1) is located in the middle reaches of the Yellow River and the upper reaches of the Yangtze River of the eastern part of northwest China, and it falls between latitudes 31°42′ and 39°35′N, and longitudes 105°29′ and 111°15′E. The area is 2.058×10^5^ km^2^, extending about 880 km from north to south and 160 to 490 km from east to west. The whole province from north to south can be divided into four agro-ecological zones, which include the Loess Plateau area of northern Shaanxi, the Weibei dry plateau, the Guanzhong irrigated area and the Qin-Ba mountain area of southern Shaanxi; the previous three regions belong to the Loess Plateau and in this study they are abbreviated as North, Weibei, and Guanzhong, respectively. The Loess Plateau region in China, covers five provinces (including Shaanxi province), stretches over an area of 0.62 million km^2^, and consists of typical semiarid and arid areas with rainfed farming [17]–[18]. Winter wheat is planted in the regions of Weibei and Guanzhong, while summer maize is planted in the Guanzhong region and spring maize in the North and Weibei regions. Main soil types and climatic conditions in the different regions are shown in Table 1.

**Table 1:** Main soil types and climatic conditions in the different regions.

Region	Main soil types	Annual mean temperature (°C)	Annual precipitation (mm)
North	Castanozems, Sierozems, Loess soils	8∼11	275∼590
Weibei	Black loess soils, Loess soils	9∼13	530∼630
Guanzhong	Cinnamon soils	10∼14	600∼720

### Data sources

**Figure 2:**
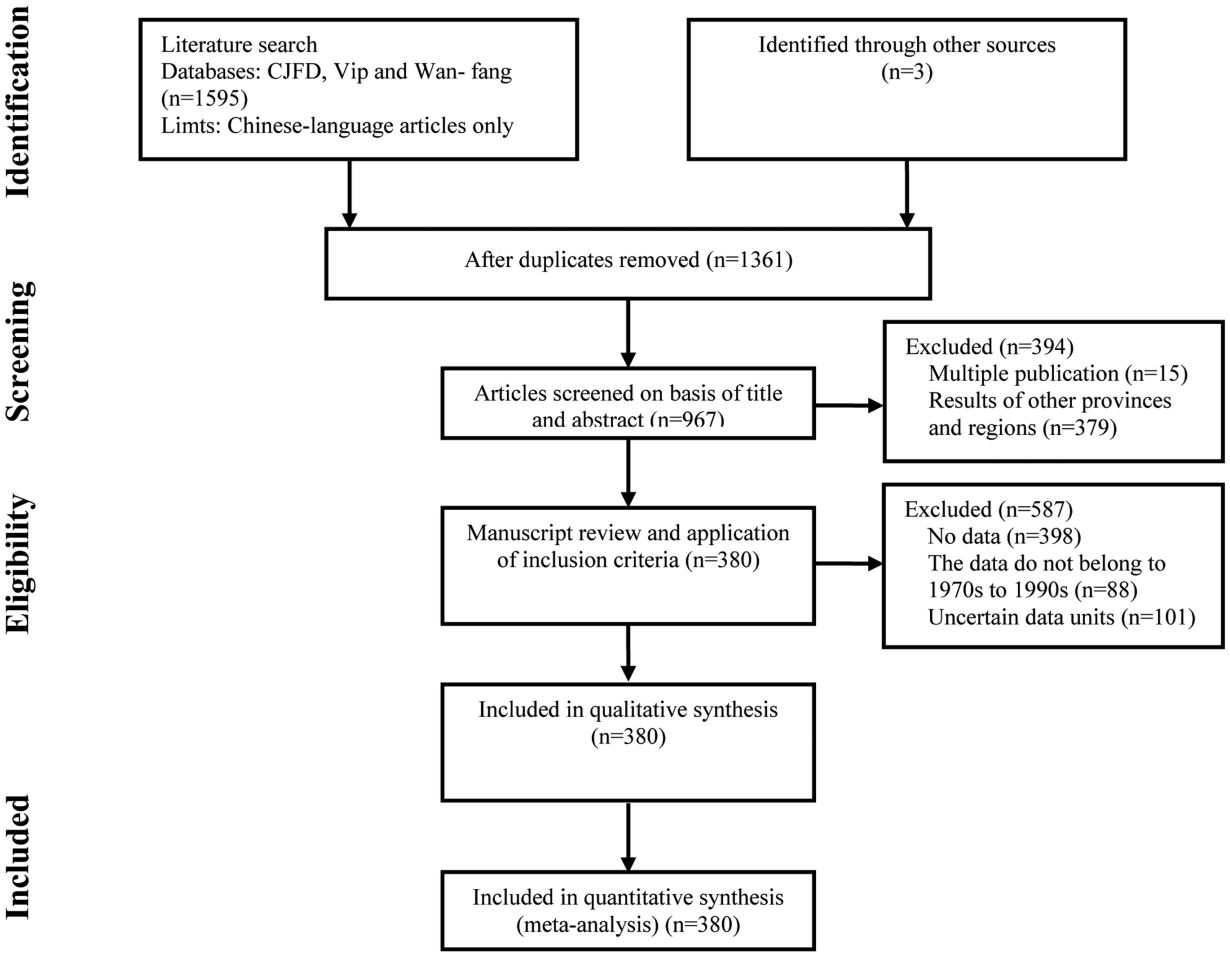
The screening process and results for literature from the 1970s to the 1990s.

The data from the 1970s to the 1990s was extracted from 380 published papers reporting household fertilization and soil fertility in the study area; the screening process and results are shown in Figure 2. Data from the 2000s was collected from the project “soil testing and formulated fertilization in Shaanxi province during the years 2005 to 2009.”

### Statistics

The data were analyzed by EXCEL software. In this study, we used the following equations to analyze the soil nutrient balance and partial factor productivity (PFP) of fertilizer:
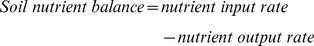
(Eq 1)where the nutrient input rate represents chemical fertilizer input, and the nutrient output rate represents amounts extracted in crop products and above ground biomass;
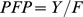
(Eq 2)where Y represents crop yields, and F represents chemical fertilizer input.

## Results

### Spatial and temporal variations of chemical fertilization and yields of wheat and maize at the farmers' level in different regions of Shaanxi province

**Figure 3:**
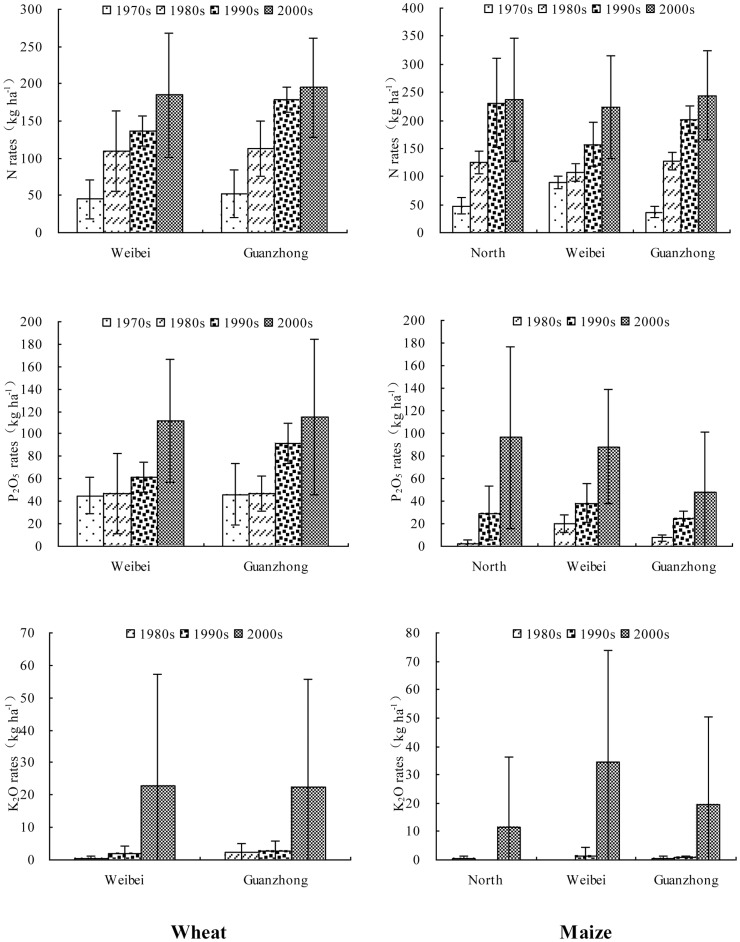
Variations of chemical fertilization for wheat and maize at the farmers' level in different regions of Shaanxi province (error bars show standard deviations).

The average chemical fertilizer NPK inputs for both wheat and maize at the farmers' level increased for decades in the different regions (Figure 3). In the Weibei and Guanzhong regions, chemical fertilizer N inputs for wheat in the 1970s were 45 kg ha^−1^ and 52 kg ha^−1^, respectively, and in the 2000s they increased to 185 kg ha^−1^ and 195 kg ha^−1^, respectively. In these two regions, chemical fertilizer P_2_O_5_ inputs were 45 kg ha^−1^ and 46 kg ha^−1^ in the 1970s and they increased to 112 kg ha^−1^ and 115 kg ha^−1^ in the 2000s. In the 1980s, farmers started to use the chemical fertilizer K_2_O for wheat, which was increased from 0.5 kg ha^−1^ and 2.3 kg ha^−1^ to 22.8 kg ha^−1^ and 22.5 kg ha^−1^, respectively, during the 1980s to the 2000s in the two regions. For maize in the North, Weibei and Guanzhong regions, chemical fertilizer N inputs were 48 kg ha^−1^, 89 kg ha^−1^ and 36 kg ha^−1^ and they increased to 237 kg ha^−1^, 223 kg ha^−1^ and 244 kg ha^−1^, respectively, from the 1970s to the 2000s. Unlike wheat, from the 1980s onward farmers were awarded for using the chemical fertilizers P_2_O_5_ and K_2_O for maize, and their use has increased greatly.

**Figure 4:**
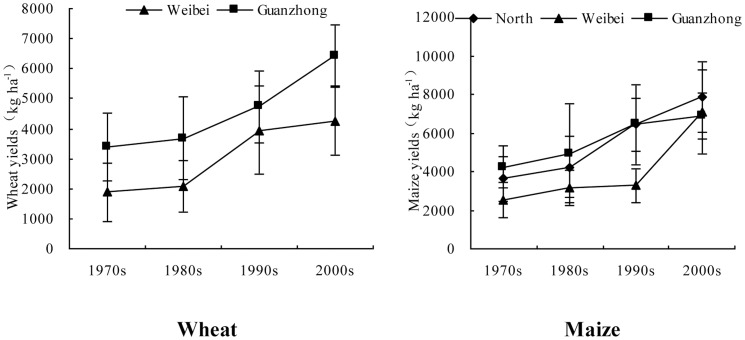
Variations of yields for wheat and maize at the farmers' level in different regions of Shaanxi province (error bars show standard deviations).

In accordance with increased chemical fertilizer NPK inputs (Figure 3), the average yields of wheat and maize showed increasing trends in the different regions over the four decades (Figure 4). In the Weibei and Guanzhong regions, from the 1970s to the 2000s, yields of wheat changed from 1883 kg ha^−1^ and 3377 kg ha^−1^ to 4269 kg ha^−1^ and 6437 kg ha^−1^, with increase rates of 127% and 91%, respectively. In the North, Weibei and Guanzhong regions, yields of maize changed from 3636 kg ha^−1^, 2519 kg ha^−1^ and 4232 kg ha^−1^ to 7867 kg ha^−1^, 7077 kg ha^−1^ and 6886 kg ha^−1^, with increase rates of 116%, 181% and 63%, respectively, for the same period.

### Spatial and temporal variations of soil nutrient balance from the inputs and uptake on wheat and maize plots in different regions of Shaanxi province

**Figure 5:**
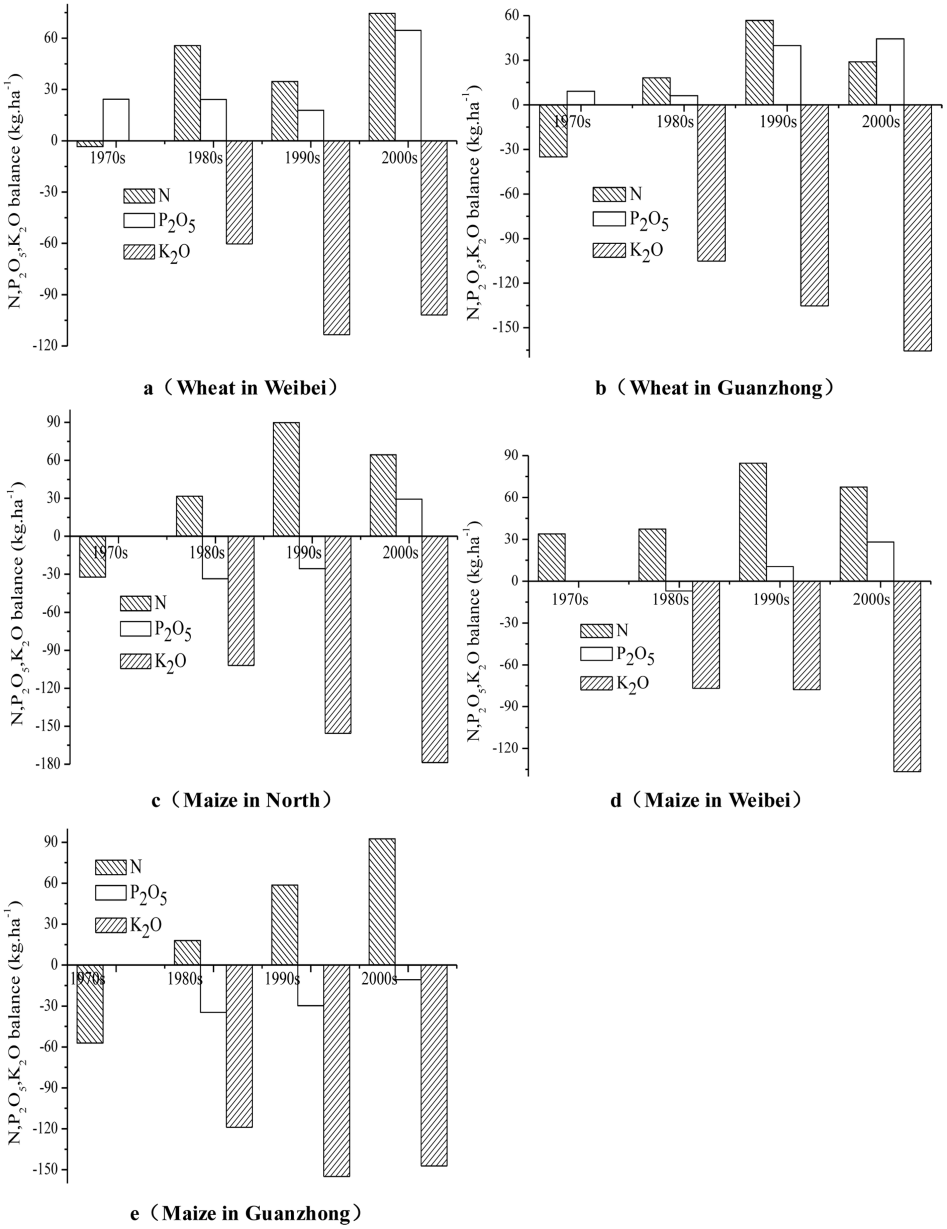
Variations of soil nutrient balance on wheat and maize plots in different regions of Shaanxi province.

Because the farmers tended not to use organic manure for cereal crops, especially from the 1980s onward, the soil nutrient inputs only include chemical fertilizers, and the nutrient uptakes include those extracted in crop products and above ground biomass. The nutrient balance was calculated as the difference between the average input and uptake (Eq. 1). Other losses, from leakage and gaseous loss, were not included in these calculations. In the 1970s, N was deficient on wheat and maize plots in the different regions (except for maize plots in the Weibei region). Then from the 1980s N was consistently at surplus levels, and it displayed an upward trend with time. In the 2000s, N surpluses on wheat plots were 74 kg ha^−1^ and 29 kg ha^−1^ in the Weibei and Guanzhong regions, respectively; meanwhile N surpluses on maize plots were 64 kg ha^−1^, 67 kg ha^−1^ and 93 kg ha^−1^ in the North, Weibei and Guanzhong regions, respectively (Figure 5).

In the Weibei and Guanzhong regions, the amount of surplus P_2_O_5_ on wheat plots increased each year from the 1970s to the 2000s, and surplus amounts increased from 24 kg ha^−1^ and 9 kg ha^−1^ to 65 kg ha^−1^ and 44 kg ha^−1^, respectively. In the North, Weibei and Guanzhong regions, P_2_O_5_ was deficient on maize plots in the 1980s; then it gradually reached surplus levels until the 2000s with the increased application of chemical fertilizer phosphorus. The balance of P_2_O_5_ on maize plots increased from −34 kg ha^−1^, −7 kg ha^−1^ and −35 kg ha^−1^ to 29 kg ha^−1^, 28 kg ha^−1^ and −11 kg ha^−1^, respectively, in the three regions from the 1980s to the 2000s. It is worth noting, that winter wheat and summer maize were in a rotation system in the Guanzhong region, so total P_2_O_5_ was in surplus in this region in the 2000s and the amount was 33 kg ha^−1^ (Figure 5).

Although farmers have been awarded for using K_2_O chemical fertilizer in recent years, the amount used was still small (Figure 3), and it was usually from compound fertilizers. So K_2_O deficiency has become more serious (Figure 5). In the 2000s, K_2_O deficiency levels on wheat plots were −102 kg ha^−1^ and −165 kg ha^−1^ in the Weibei and Guanzhong regions, respectively; meanwhile K_2_O deficiency levels on maize plots were −179 kg ha^−1^, −137 kg ha^−1^ and −147 kg ha^−1^ in the North, Weibei and Guanzhong regions, respectively.

### Spatial and temporal variations of soil fertility in different regions of Shaanxi province

**Figure 6:**
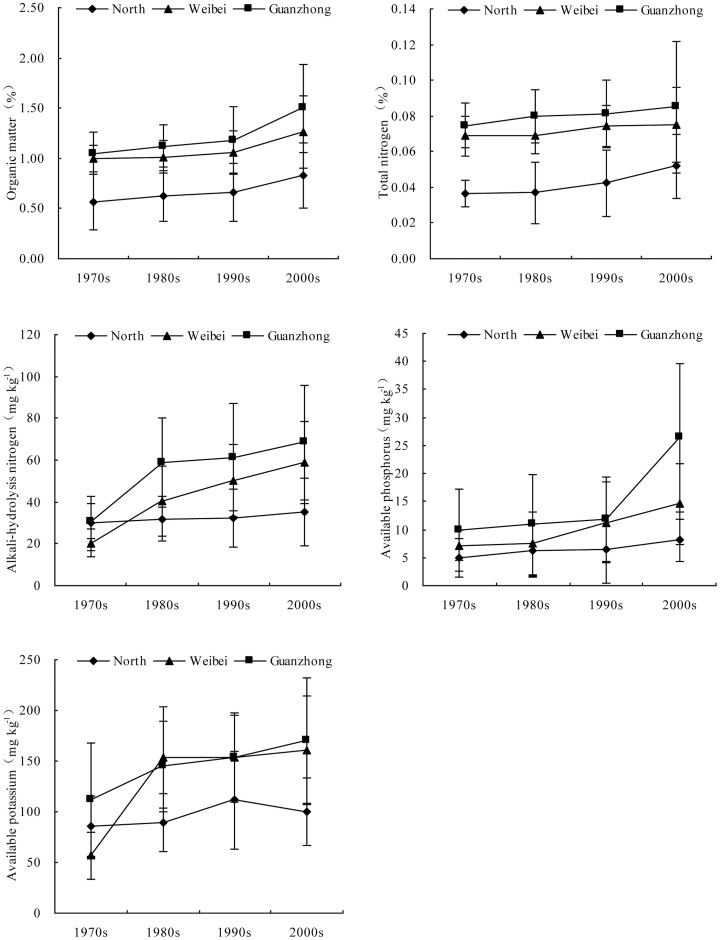
Variations of soil organic matter, total nitrogen, alkali-hydrolysis nitrogen, available phosphorus and available potassium in different regions of Shaanxi province (error bars show standard deviations).

In the different regions of Shaanxi province, soil fertility indexes, including organic matter, total nitrogen, alkali-hydrolysis nitrogen, available phosphorus and available potassium, all increased from the 1970s to the 2000s. Simultaneously, each of these five indicators increased from the north to the south during the same period (North<Weibei<Guanzhong) (Figure 6). In the North, Weibei and Guanzhong regions from the 1970s to the 2000s, organic matter varied from 0.57%, 1.01% and 1.12% to 0.83%, 1.26% and 1.50%, with increase rates of 46%, 26% and 43%, respectively; total nitrogen varied from 0.04%, 0.07% and 0.07% to 0.05%, 0.08% and 0.09%, with increase rates of 42%, 9% and 14%, respectively; alkali-hydrolysis nitrogen varied from 29.95 mg kg^−1^, 20.43 mg kg^−1^ and 30.81 mg kg^−1^ to 35.20 mg kg^−1^, 58.70 mg kg^−1^ and 68.40 mg kg^−1^, with increase rates of 18%, 187% and 122%, respectively; available phosphorus varied from 4.98 mg kg^−1^, 7.13 mg kg^−1^ and 9.90 mg kg^−1^ to 8.10 mg kg^−1^, 14.60 mg kg^−1^ and 26.40 mg kg^−1^, with increase rates of 63%, 105% and 167%, respectively; available potassium varied from 85.60 mg kg^−1^, 56.78 mg kg^−1^ and 111.75 mg kg^−1^ to 99.60 mg kg^−1^, 160.70 mg kg^−1^ and 170.40 mg kg^−1^, with increase rates of 16%, 183% and 52%, respectively.

### Relationships among fertilizer inputs, crop yields and soil fertility in different regions of Shaanxi province

Because farmers used little P and K fertilizers in the 1970s and 1980s (Figure 3), only PFP of N, NP and NPK were calculated in the study (Eq. 2). The PFP of N, NP and NPK on wheat and maize decreased from the 1970s to the 2000s as a whole in the different regions (Table 2). The PFP of N on wheat in the Weibei and Guanzhong regions were 42 kg kg^−1^ and 65 kg kg^−1^, respectively, in the 1970s, which decreased to 23 kg kg^−1^ and 33 kg kg^−1^, respectively, in the 2000s. Meanwhile the PFP of N on maize in the North and Guanzhong regions were 76 kg kg^−1^ and 118 kg kg^−1^, respectively, and they decreased to 33 kg kg^−1^ and 28 kg kg^−1^ from the 1970s to the 2000s. In the Weibei region, the PFP of N on maize changed slightly from 28 kg kg^−1^ to 32 kg kg^−1^, which resulted from the use of high N inputs relative to the other two regions (up to 89 kg ha^−1^) in the 1970s (Figure 3). This led to a low PFP of N in that period. The PFP of NP on wheat decreased to 14 kg kg^−1^ and 21 kg kg^−1^ in the Weibei and Guanzhong regions, respectively; in maize it decreased to 24 kg kg^−1^, 23 kg kg^−1^ and 24 kg kg^−1^ in the North, Weibei and Guanzhong regions, respectively. Similar to N and NP, the PFP of NPK on wheat decreased to 13 kg kg^−1^ and 19 kg kg^−1^ in the Weibei and Guanzhong regions, respectively; in maize it decreased to 23 kg kg^−1^, 20 kg kg^−1^ and 22 kg kg^−1^ in the North, Weibei and Guanzhong regions, respectively (Table 2).

**Table 2:** Variations of PFP of fertilizer on wheat and maize in the different regions (kg kg^−1^).

Crop	Fertilizer type	Region	1970s	1980s	1990s	2000s
Wheat	N	Weibei	42	19	29	23
		Guanzhong	65	33	26	33
	N+P_2_O_5_	Weibei	21	13	20	14
		Guanzhong	34	23	17	21
	N+P_2_O_5_+K_2_O	Weibei	21	13	20	13
		Guanzhong	34	23	17	19
Maize	N	North	76	34	28	33
		Weibei	28	30	21	32
		Guanzhong	118	39	32	28
	N+P_2_O_5_	North	76	33	25	24
		Weibei	28	25	17	23
		Guanzhong	118	37	29	24
	N+P_2_O_5_+K_2_O	North	76	33	25	23
		Weibei	28	25	17	20
		Guanzhong	118	37	29	22

**Figure 7:**
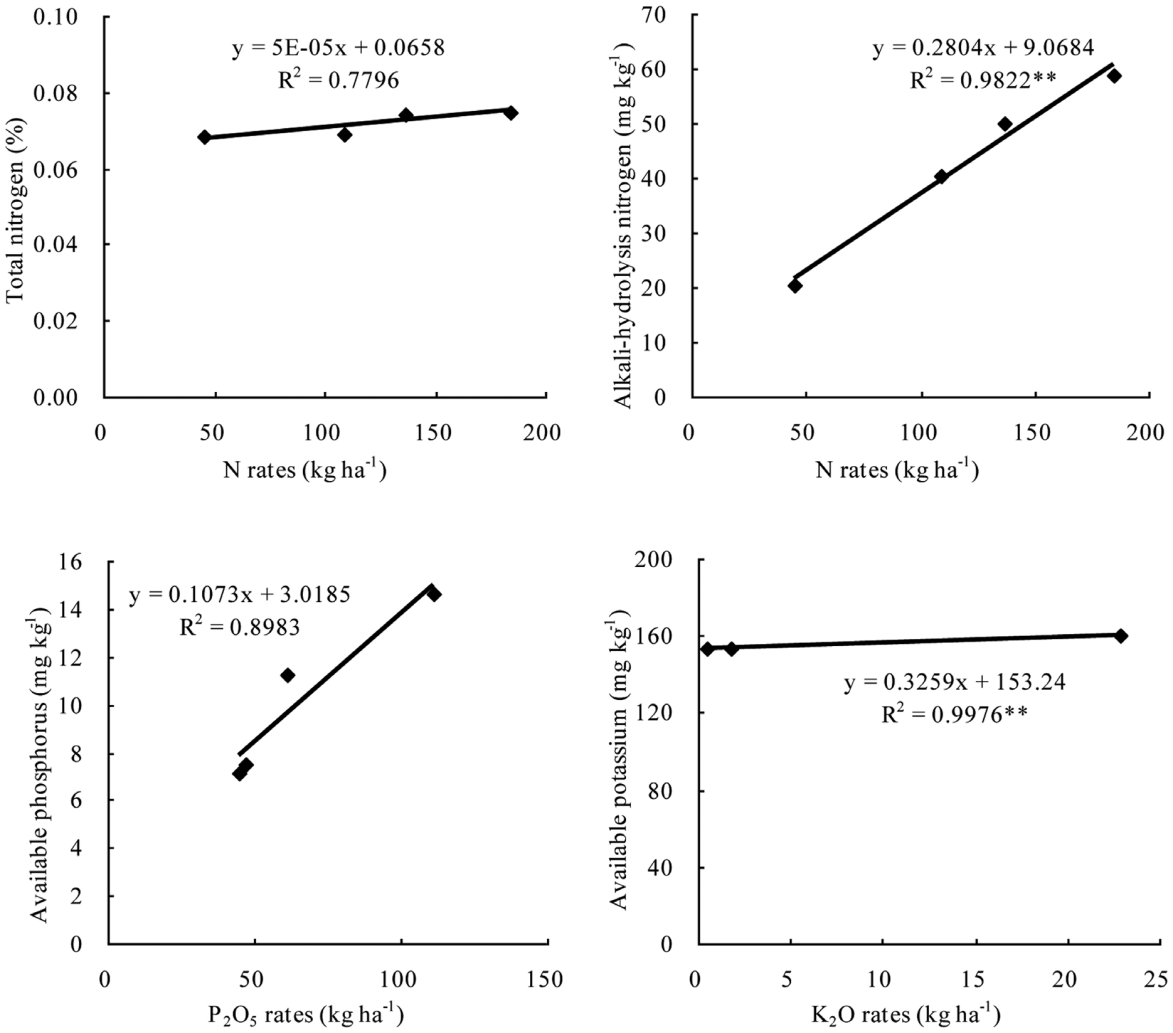
Relationships between N rates and total nitrogen, N rates and alkali-hydrolysis nitrogen, P_2_O_5_ rates and available phosphorus and K_2_O rates and available potassium on wheat plots in the Weibei region of Shaanxi province. **Significance level: P<0.01.

**Figure 8:**
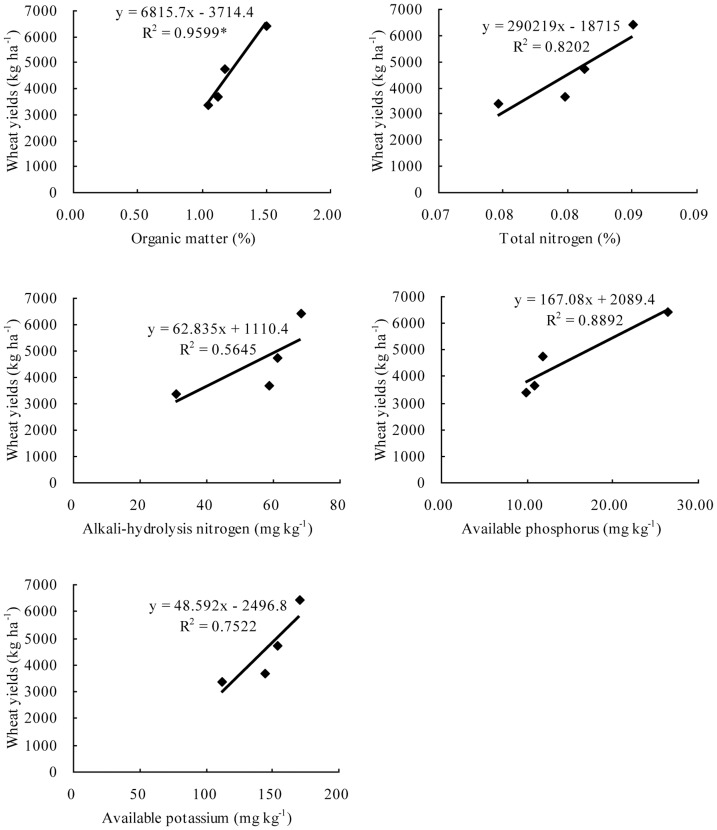
Relationships between wheat yield and soil organic matter, total nitrogen, alkali-hydrolysis nitrogen, available phosphorus and available potassium in the Weibei region of Shaanxi province. *Significance level: P<0.05.

In order to find relationships among soil fertility, crop yields and fertilizer rates, we used the Weibei region as an example. The values of fertilization, crop yields and soil fertility did not have one to one correspondence from the 1970s to the 1990s, so their mean value from each period was examined (Figures 7 and 8). Although the sample size was small and some relationships did not reach significant levels, with the increase in N fertilizer inputs, soil total nitrogen and alkali-hydrolysis nitrogen both increased. P fertilizer increased soil available phosphorus and K fertilizer increased soil available potassium significantly (Figure 7). At the same time, soil organic matter, total nitrogen, alkali-hydrolysis nitrogen, available phosphorus and available potassium all had positive impacts on wheat yields (Figure 8).

## Discussion

Fertilizer use efficiency of both wheat and maize decreased from the 1970s to the 2000s as a whole in the Loess Plateau of Shaanxi (Table 2), which was consistent with national trends. Nitrogen fertilizer, phosphorus fertilizer and potassium fertilizer use efficiencies were 30–35%, 15–20% and 35–50%, respectively, from 1981 to 1983, and the average values decreased to 28%, 12% and 32% on cereal crops by 2001 to 2005 in China [19]. This suggested that the effect of chemical fertilizers on increasing grain production had diminished. The PFP of N on wheat in the Weibei and Guanzhong regions decreased to 23 kg kg^−1^ and 33 kg kg^−1^, respectively, and the PFP of N on maize in the North, Weibei and Guanzhong regions were 33 kg kg^−1^, 32 kg kg^−1^ and 28 kg kg^−1^, respectively, in the 2000s (Table 2). Zhang et al. [19] reported average PFP values of N for wheat and maize of 43 kg kg^−1^ and 52 kg kg^−1^, respectively, in China. Dobermann and Cassman [20] reported a global average PFP of N for cereals of 44 kg kg^−1^. This indicated that nitrogen use efficiency on wheat and maize in the Loess Plateau of Shaanxi was much lower than the current national and global levels. Excessive fertilization has been the main reason for low fertilizer use efficiency in China [19]. In addition, Liu et al. [21] reported that in agro-ecosystems, surplus N increased from 1978 to 2005 throughout the country, and our findings on the Loess Plateau were consistent with this trend. For example, in the 2000s, chemical fertilizer N inputs on maize were 237 kg ha^−1^, 223 kg ha^−1^ and 244 kg ha^−1^ in the North, Weibei and Guanzhong regions, respectively (Figure 3); meanwhile N surpluses on maize plots were 64 kg ha^−1^, 67 kg ha^−1^ and 93 kg ha^−1^, respectively, in the three regions (Figure 5). This indicated that excessive N fertilization was a serious problem in the Loess Plateau, and the same phenomenon has been reported many times in China, for example, in Beijing [16], [22], Shandong [1], [23]–[25], and Jiangsu [26]–[27]. Excessive N fertilization not only wastes resources, but also leads to many serious environmental problems [28]–[31] including nitrate pollution of groundwater [32]–[37], eutrophication of surface water [38]–[39], greenhouse gas emissions and other forms of air pollution [40]–[42], acid rain [43]–[46], soil acidification [36], [47]–[50] and so on. On the other hand, a lower fertilization rate does not necessarily reduce crop yields [51]. Many studies have shown that reducing the current N application rates by 30 to 60% could increase N fertilizer efficiency, while still maintaining crop yields and substantially reducing N losses to the environment [31], [52]–[53].

Like nitrogen, phosphate fertilizer inputs (Figure 3), P surpluses (Figure 5) and soil available phosphorus levels (Figure 6) all increased in the last 40 years on the Loess Plateau in Shaanxi. Similar results have been noted in north China and all over the country [25], [54]. Yang et al. [55] reported that maintaining soil available phosphorus at a relatively high level requires a P application rate of about 80 kg ha^−1^ yr^−1^ in winter wheat/summer maize rotation systems in the Guanzhong region. Our results showed phosphate fertilizer inputs of up to 163 kg ha^−1^ in winter wheat/summer maize rotation systems in this region in the 2000s (Figure 3). This indicated that P fertilization was also excessive, which not only wasted resources but also led to many serious environmental problems [28]–[31]. Phosphate fertilizer production consumes more than 80% of the phosphate rock resources [56], but phosphate rock resources are limited and high grade material is in short supply [57]. In addition, the phosphate fertilization utilization ratio of the main crops ranged from 7% to 20%. It averages 12% in China [19], which has led to phosphorus accumulation in the soil, increasing the risk of non-point source pollution from surface runoff [58]. Agricultural non-point source pollution has become an increasingly serious problem in China, primarily because it leads to eutrophication.

In spite of increased K fertilizer inputs on wheat and maize in recent years (Figure 3), the soil K balance has become increasingly negative (Figure 5) and soil available potassium has increased (Figure 6) in the last 40 years. This phenomenon was previously reported in northwest and north China [55], [59]–[60]. Evidently, K fertilizer application was not the only source of K absorbed by crops. The primary sources of K for crops were weathering of parent materials [60]–[61], release of K into the soil from increased soil organic matter and changes in soil pH [61]. Yang et al. [55] found that soil organic matter content in all treatments (including those without fertilizer) significantly increased over time and soil pH dropped from the initial value of 8.65 to 8.58 from 1991 to 2010 during long-term field trials in the Guanzhong region. Our results showed that in the North, Weibei and Guanzhong regions soil organic matter increased from 0.57%, 1.01% and 1.12% to 0.83%, 1.26% and 1.50%, respectively, from the 1970s to the 2000s (Figure 6). The average soil pH has declined 0.5 units with the overuse of N fertilizer in the past two decades in China [62]. Li et al. [63] reported that the soil pH decreased from the initial value of 8.76 to 8.56 from 1992 to 2008 during long-term field trials in the North region. There may be other mechanisms involved, for example, crops might draw on K in the deeper soil layers or from the non-exchangeable pool. The contribution of K from the subsoil could be considerable [64]. Witter and Johansson [65] found that 41–47% of the K was from the subsoil for green manure crops. Many studies have shown that crops use non-exchangeable K [66]–[67]. Decreases in the abundance of non-exchangeable K with simultaneous increases in exchangeable and water-soluble K concentrations suggest that much of the K taken up by crops comes from non-exchangeable species via solution and exchangeable phases in a way that establishes and maintains the equilibrium between various forms of K in the soil [66].

Fertilizer rates had a large effect on soil fertility. With the increase in N fertilizer inputs, both soil total nitrogen and alkali-hydrolysis nitrogen increased; P fertilizer increased soil available phosphorus and K fertilizer increased soil available potassium significantly in the Weibei region (Figure 7). It has been reported that after 25 years of N fertilization, soil organic carbon and total nitrogen had increased by 18% and 26%, respectively, from 1984 to 2009 in the Weibei region [18]. Cai and Hao [68] also found that accumulation of soil nitrogen initially increased and then decreased with increasing nitrogen, and total nitrogen and alkali-hydrolysis nitrogen content reached the highest value or the second highest value of 135 kg ha^−1^ on wheat plots in the Weibei region, which was in accordance with findings in northwest and north China by Li et al. [63] and Lin et al. [69]. Through long-term field experimentation on the Loess Plateau in Shaanxi, Li et al. [63] and Hao et al. [70] found that with increases in P fertilizer inputs, soil available P increased significantly. Similar results have been obtained in northeast and northwest China by Geng et al. [71] and Zhao et al. [72], and also in America by Griffin et al. [73]. In addition, Li et al. [74] found that with increased K fertilizer inputs, soil available K increased significantly in a long-term field experiment on the Loess Plateau. Furthermore, many studies in this area have shown that on the basis of N and P fertilizer application, long-term K fertilizer application can increase soil available K and grain yields [75]–[76].

Our research also found that soil fertility had a positive impact on crop yields (Figure 8). Zhou et al. [77] revealed that soil organic carbon and total nitrogen concentrations had a significant effect on crop yields in the semi-arid Loess Plateau by long-term experimentation. Higher yields without fertilizer were generally obtained in soils with higher average soil organic matter concentrations. For example, yields without fertilizer <4000 kg ha^−1^ were obtained with average soil organic matter concentrations of 1.41% for winter wheat and 1.46 for summer maize. In contrast, average soil organic matter concentrations were 1.69% for winter wheat and 1.61% for summer maize for plots with yields>6000 kg ha^−1^ without fertilizer in north China [78]. Gong et al. [79] also found that the contribution percentage of basic soil productivity to wheat yield was significantly correlated with soil organic carbon, total nitrogen, available nitrogen, available phosphorus and available potassium in long-term soil fertility experiments in north China. Similar results have been obtained in other parts of mainland China [80], indicating that inherent soil productivity contributed to the substantial increase in China's crop yields.

In addition, although the use of chemical fertilizers to supplement NPK nutrients in the soil is important, many researchers at home and abroad reported that the application of chemical fertilizer in combination with organic manure is helpful in maintaining soil fertility (especially soil organic carbon) and buffering capacity, and in reducing NO_3_-N accumulation in the soil, while maintaining high soil productivity [4], [81]–[87].

## Conclusions

From the 1970s to the 2000s in the North, Weibei and Guanzhong regions of the Loess Plateau in Shaanxi province, chemical fertilizer NPK inputs and yields of wheat and maize increased at the farmers' level. In the 1970s, N was deficient on wheat and maize plots in the different regions; thereafter N was in surplus. In the same way, P gradually changed from deficit to surplus levels. In addition, soil organic matter, total nitrogen, alkali-hydrolysis nitrogen, available phosphorus and available potassium increased over the same period. However, K deficiencies became more and more severe. The PFP of N, NP and NPK on wheat and maize all decreased from the 1970s to the 2000s as a whole. With the increase in N fertilizer inputs, both soil total nitrogen and alkali-hydrolysis nitrogen increased; P fertilizer increased soil available phosphorus and K fertilizer increased soil available potassium significantly. At the same time, soil organic matter, total nitrogen, alkali-hydrolysis nitrogen, available phosphorus and available potassium all had positive impacts on crop yields. In order to promote food safety and environmental protection, farmers should be encouraged to assess their fertilizer needs carefully. Many can reduce nitrogen and phosphate fertilizer inputs significantly and increase potassium fertilizer and organic manure on cereal crops.
